# Psychosocial safety climate (PSC) at middle management level in the healthcare sector: A contribution to the Italian validation of psychosocial safety climate-4

**DOI:** 10.3389/fpsyg.2022.1046286

**Published:** 2022-11-28

**Authors:** Alice Fattori, Anna Comotti, Lorenzo Bordini, Maureen F. Dollard, Matteo Bonzini

**Affiliations:** ^1^Department of Clinical Sciences and Community Health, University of Milan, Milan, Italy; ^2^Occupational Medicine Unit, IRCCS Maggiore Policlinico Hospital Foundation, Milan, Italy; ^3^PSC Global Observatory, Centre for Workplace Excellence, Justice and Society, University of South Australia, Adelaide, SA, Australia

**Keywords:** psychosocial safety climate (PSC), burnout, job satisfaction, job demands-resources model (JD-R model), midlevel leadership, Effort-Reward Imbalance (ERI), hospital worker

## Abstract

**Introduction:**

Psychosocial safety climate (PSC) refers to workers’ shared perceptions of organizational policies, practices and procedures for the protection of psychological health and safety. PSC offers a multilevel organizational approach that expands traditional models of workplace stress, giving a more comprehensive understanding of occupational health and safety issues. Although considerable research on psychosocial risks in the healthcare sector has been conducted, few studies have explored the role of PSC among healthcare workers at middle management level. Additionally, no validated version of PSC is available in Italian language. The aim of this study is to contribute to the validation of the Italian 4-item version of the PSC and to explore this theory within the Job Demands-Resources model (JD-R) among a sample of Italian healthcare workers by testing PSC at the middle management level.

**Methods:**

We used cross-sectional data from 276 employees working in 17 different wards in a large Italian hospital. Intra-class coefficient (ICC) coefficient and agreement index were used to test PSC as a climate construct (data nested to hospital ward level). We performed hierarchical linear models to test mediation and moderation effects.

**Results:**

The Italian version of PSC-4 proved to have good psychometric properties and confirmed its role as a group-level construct (α = 0.84; ICC = 0.16). Multilevel random coefficient models showed PSC was associated with Job demands (Effort: *B* = −0.36, SE = 0.07; Emotional demands: *B* = −0.03, SE = 0.01) and Job resources (Reward: *B* = 1.16, SE = 0.01; Physical work environment: *B* = 0.06, SE = 0.01). Results confirmed the indirect effect of PSC on Psychological (Burnout) and Occupational health (Job satisfaction) outcomes supporting the role of Job resources and Job demands as mediators. The multilevel analysis did not find a significant interaction terms between PSC and Job demands on Burnout therefore the moderation hypothesis was not supported.

**Discussion:**

The Italian version of PSC-4 is a valid tool to evaluate PSC. These findings sustain the multilevel framework of PSC and the significant role played by mid-leaders in both the health impairment and motivational path. Further studies should explore the buffering effect of PSC at higher baseline levels as well as the adoption of PSC as a target for occupational health intervention the Italian context.

## Introduction

There is a large amount of literature indicating how healthcare workers are exposed to considerable psychosocial risk factors, such as high workload, emotional demands, lack of supervisor support, understaffing, and aggressive behaviors (European Agency for Safety and Health at Work, [Eu-Osha], 2014; West et al., 2018; Eurofound, 2020). These risk factors can generate work-related stress which can over time lead to burnout (Bria et al., 2012; Molina-Praena et al., 2018; Patel et al., 2018). Research has found that burnout is a common experience among health professionals with a potential detrimental impact on individual and organizational health and significant economic cost related to turnover and reduced clinical hours (Hakanen and Schaufeli, 2012; Aiken et al., 2013; Han et al., 2019).

In the last few decades, researchers developed different workplace stress theoretical models that have brought significant insight into the relationship between job design and workers’ health and performance (Daniels et al., 2014). The Job Demands and Resources model (JD-R) is one of the most cited models of workplace stress that has proven to be an effective framework for describing the underlying mechanisms between organizational conditions and their outcomes on health and job performance (Bakker and Demerouti, 2007, 2017). According to this model, all the organizational characteristics that require physical and psychological efforts (namely, “job demands”) have been identified as main causes of adverse health outcomes, such as burnout (*the health impairment process*), whereas the physical, social, or organizational aspects of the job that act as driving forces to support employees in achieving working goals and reducing job demands (namely, “job resources”) result in high levels of motivation and excellent job performance (*the motivational process*).

In recent years, scholars have been pointing out that workplace stress theories could benefit from combining the job conditions, traditionally considered at the individual level, with a multilevel organizational approach in order to achieve a more comprehensive understanding and management of occupational health and safety issues (Morgeson et al., 2010; Zadow and Dollard, 2016). In this respect, psychosocial safety climate (PSC) theory is an emerging and multilevel approach that has been proposed as an extension of JD-R and other current models of workplace stress (Dollard and Bakker, 2010). PSC is a specific dimension of organizational climate that reflects employees’ shared perceptions on “policies, practices and procedures for the protection of workers psychological health and safety” (p. 580; Dollard and Bakker, 2010). Psychosocial safety climate has four domains comprising (1) management support and commitment for stress prevention through involvement and commitment, (2) management priority to psychological health and safety versus productivity goals, (3) communication between the organization and employees on psychological health and safety issues, and (4) organizational participation and involvement in protecting workers’ psychological health.

As a primary function, this theory proposes that PSC as an organizational-level construct predicts job design and, consequently, both job demands and resources, as their level and type result from management priorities and values for occupational health and safety. Additionally, PSC has a secondary function as it moderates the effects of job demands on psychological health outcomes (Dollard and Bakker, 2010; Law et al., 2011; Dollard et al., 2012b). Several studies so far have supported the dual function of PSC as a main effect (“cause of the causes”) and moderator in the occupational stress process (Idris and Dollard, 2016).

Evidence also supports psychosocial safety climate as a multilevel theory combining organizational and individual levels: Although few studies adopted PSC at the individual level, most research confirmed psychosocial safety climate as an indicator of the shared perception at group/teams level reflecting an organization characteristic more than an individual feature (Dollard et al., 2012a; Idris and Dollard, 2016).

**FIGURE 1:**
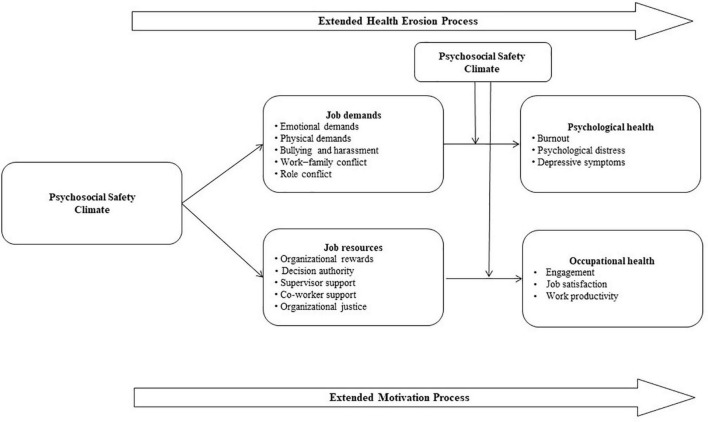
Psychosocial safety climate theoretical framework within the JD-R model.

The psychosocial safety climate theoretical framework provides an extension of JD-R theory as PSC precedes both job demands and job resources as defined in the JD-R model and moderates the impact of job demands on psychological health; this framework extends both the health impairment and the motivational pathway as job demands and job resources mediate the relationship between PSC and psychological and occupational health outcomes (Figure 1).

Psychosocial safety climate can be measured through a 12-item tool (PSC-12) that reflects the four main theoretical domains of PSC, i.e., Management Commitment and Support, Management Priority, Organizational Participation, and Organizational Communication. PSC-12 has been used across different occupations and organizations, and different studies confirmed the psychometric properties of PSC (Hall et al., 2010; Law et al., 2011; Idris et al., 2012). Recently, a PSC-12 shorter version with four items (an item for each PSC domain) was developed and preliminary research demonstrated good validity and reliability (Dollard, 2019; Berthelsen et al., 2020). Both PSC-12 and PSC-4 items can be addressed to evaluate workers’ perception of psychosocial safety climate at a higher organizational level (i.e., top management commitment, support, priorities) or a middle management level (i.e., commitment, support, priorities of those who lead teams within organizations); indeed, although PSC reflects organization’s and top management’s strategic imperatives on employees’ psychological health, midlevel leaders play also a role in shaping the climate within their teams (Loh et al., 2021; Parent-Lamarche and Biron, 2022).

To our knowledge, no studies have tested so far the PSC theory in the Italian context neither is Italian validation of PSC tool available.

### The current study

The aim of this study was to validate the Italian version of PSC-4 by testing the PSC theory among a sample of Italian healthcare workers. Similar to previous research, we tested PSC theory within the JD-R model framework (Zadow et al., 2019): We used “Effort” and “Emotional demand” as Job demands, “Reward” and “Physical work environment” as Job resources, “Burnout” as a Psychological health outcome, and Job satisfaction as an Occupational health outcome.

**FIGURE 2:**
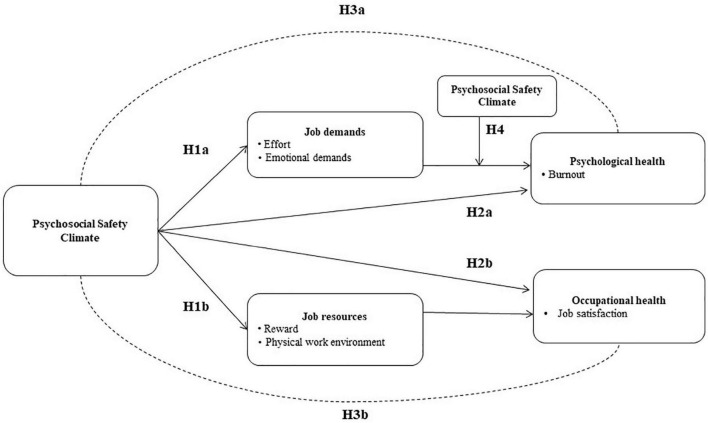
Study model.

Consequently, we generated the following hypothesis (Figure 2).

Hypothesis 1. PSC will be negatively related to Job demands (Effort, Emotional demands: H1a) and positively related to Job resources (Reward, Physical work environment: H1b);

Hypothesis 2. PSC will be negatively associated with Psychological health (Burnout: H2a) and positively associated with Occupational health (Job satisfaction: H2b);

Hypothesis 3. Job demands (Effort, Emotional demands) will mediate the relationship between PSC and Psychological health (Burnout; H3a) and Job resources (Reward, Physical work environment) will mediate the relationship between PSC and Occupational health (Job satisfaction: H3b);

Hypothesis 4. PSC will moderate the relationship between Job demands (Effort, Emotional demands) and Psychological health (Burnout). At high levels of PSC, the negative relationship between demands and health should be reduced.

## Materials and methods

### Participants and data collection

A cross-sectional survey was carried out in 17 different hospital wards of a large Hospital of Northern Italy. The survey was conducted by occupational health psychologists and occupational physicians within an occupational health risk assessment as required by the Italian law. All employees currently active in the hospital wards at the time of data collection were involved in the survey on a voluntary basis. The only exclusion criterion was a job seniority of less than six months as we reasoned that some experience with the organization was required to assess the work environment. Before the assessment phase, informative meetings with managers, employees, and workers representatives took place in each ward, with the purpose of clarifying the objectives and procedures as well as increasing subjects’ commitment with the survey. As requested by the Italian regulation, employees who participated read and signed consent and privacy forms. All data were collected in the presence of psychologists/physicians in charge of the survey.

### Psychosocial safety climate-4 translation

The PSC-4 scale was translated following the back-translation criteria (Hambleton, 2005). As a first step, an occupational health psychologist with fluent English translated the four questions into Italian; the questions were then back-translated into English by a bilingual academic psychologist with considerable expertise in the research field of occupational health psychology. Subsequently, the translations were compared to reach consensus; as no changes were requested, the translators agreed on the final version which was then submitted to two expert psychologists to evaluate item clarity.

As a final step, the translators consulted the authors of PSC-4 to gather a better understanding of the management levels which potentially address the PSC evaluation (e.g., senior management, middle management). The Italian version of PSC-4 is shown in Appendix 1.

### Measures

Participants completed a self-administered questionnaire collecting sociodemographic and occupational data (age, gender, occupational role, job seniority, and job shifts). A specific item investigated how many episodes of workplace violence, both verbal and behavioral, had been experienced by workers during their work experience in this hospital (possible answers were *none, less than 3, between 3 and 10, between 11 and 20*, and *more than 20*).

*Psychosocial safety climate*. The four-item version PSC (Dollard, 2019) investigates workers’ perceptions on PSC-4 domain through a five-point scale ranging from 1 (strongly disagree) to 5 (strongly agree). For this study, we evaluated PSC perceived at the middle management level (i.e., PSC at the hospital ward level); consequently, the term “*Senior management*” was replaced with “*My ward manager*s” and “*My organization*” was replaced with “*My hospital ward.”* The total sum score ranges from 4 to 20.

*Job demands.* We measured the Emotional demands through the six-item scale by Bakker et al. (2003); example items are: “*Does your work put you in emotionally upsetting situations?*”’ and “*Are you confronted with demanding patients?*,” with a five-point rating scale ranging from 1 (never) to 5 (always). We adapted the scale to the healthcare context by replacing the generic reference to “clients/customers” with the more specific one of “patients.” We assessed workers’ Effort through the five-item Effort subscale of the Effort–Reward Imbalance Questionnaire (Siegrist, 1996); this subscale investigates the demanding aspects of the work environment (i.e., four items for quantitative and qualitative load and one item measuring the increase in total load over time) with a response scale allocating a value equal to 1 if the situation expressed in the item does not describe worker’s experience, values ranging from 2 to 5 according to the degree of stress caused by the situation. The total sum score ranges from 5 to 25, with higher scores indicating more stressful demands.

*Job resources.* Organizational Rewards were measured using the 11-item Reward subscale from the Effort–Reward Imbalance Questionnaire (Siegrist, 1996). This scale considers (a) financial reward, (b) esteem reward, and (c) reward related to promotion prospects (career) and job security. Answering and scoring procedures are the same as for the Effort subscale, with a total score from 11 to 55 (higher scores represent higher levels of rewards). Physical work environment was assessed with eight items adapted from previous research on ergonomic and working condition (see Norman, 2005); this scale evaluates workers’ satisfaction with physical exposure to air quality, furniture and equipment, noise pollution, facilities for work breaks, and changing rooms, on a five-point scale ranging from 1 (very dissatisfied) to 5 (very satisfied). A total mean score (1–5) is calculated.

*Psychological health.* Burnout was assessed with the Copenhagen Burnout Inventory (CBI; Kristensen et al., 2005). The CBI measures personal burnout, work-related burnout, and client-related burnout with three different scales; for this study, we considered the work-related burnout scale from the Italian version (Fiorilli et al., 2015; Sestili et al., 2018). Example items are: “*Do you feel worn out at the end of the working day?*” and “*Do you feel that every working hour is tiring for you?*”; scores of 100, 75, 50, 25, and 0 are attributed to “Always,” “Often,” “Sometimes,” “Seldom,” and “Never/almost never,” respectively. A total scores above 50 suggest a risk for burnout.

*Occupational health*. Job satisfaction was measured using the General Job Satisfaction scale from the Standard Shiftwork Index (Barton et al., 2007). It is a five item measure of the degree to which workers are satisfied with their job with a seven-point response scale ranging from “I strongly disagree” (scored 1) to “I strongly agree” (scored 7).

### Statistical analysis

Reliability and internal consistency of PSC-4 were assessed using Cronbach’s alpha. Preliminary analyses investigated the relation between PSC and other variables: *t*-test or one-way ANOVA tested the differences in PSC scores by subgroups (sociodemographic information, occupational role, shift work, number of aggressive episodes experienced); for continues variables (job demands, job resources, and outcomes), we computed individual and aggregate (by hospital ward) correlations with PSC, testing their significant difference from zero.

A group (hospital ward)-level analysis was performed to assess PSC as a climate construct. The intra-class coefficient (ICC) and the agreement index rwg (James, 1982) were used to compute the percentage of variance explained by groups and to assess the homogeneity in the test responses within hospital wards, respectively. According to the first index, a value of ICC equal to 0.12 is recommended in organizational research (James, 1982), while for the agreement index the cut-off of 0.70 represents the sufficient homogeneity of PSC within groups (Bliese, 2000).

Several methods for testing multilevel mediation have been proposed; Preacher et al. (2010) collected them in a general framework called multilevel structural equation model (MSEM). Such approach includes the method of Bauer et al. (2006), which was suitable for our scenario as all the study variables were measured at level 1. According to this method, we computed the average indirect effect (i.e., the portion of the effect of the predictor variable—PSC—on the outcome transmitted through the mediator variable) by multiplying the fixed-effect coefficients a*b and adding the covariance of their random effects (where a and b indicate the effect of the predictor variable on mediator and the effect of the mediator on the outcome, respectively). The estimation of a and b and of the direct effect c was conducted using random coefficient models. Confidence intervals of the indirect effect were produced using the Monte Carlo method (Bauer et al., 2006; Selig and Preacher, 2008).

Moderation analysis was preliminary explored though the graphical representation of the relationship between Job demands and Psychological health (Burnout) by different levels of PSC. To formally test moderation (H4), we added the interaction term (referred to PSC and Job demands) to the multilevel model on Burnout.

## Results

Two hundred and seventy-six employees from 17 different hospital wards took part in the study; the participation rate was high as it ranged from 71 to 97%. Workers unable to participate were mainly on sick leave or absent from work for different reasons during the data collection; to our knowledge, only three workers expressed their unwillingness to be involved in the study. Internal consistencies of all the scales were considered as acceptable (Cronbach’s alpha > 0.60).

### Psychosocial safety climate-4 psychometric properties

Cronbach’s alpha equal to 0.84 (CI 0.81, 0.87) suggested very good reliability of the test. Item responses distribution showed that percentages of “strongly disagree” and “disagree” responses ranged from 48 to 74%, resulting in an overall low perception of psychological safety climate (Table 1). High correlations between PSC-4 items and the total test score confirmed the goodness of the test.

**TABLE 1:** Distribution of PSC-4 items responses in percentage, Cronbach’s alpha if the item was deleted, and correlations with test score.

PSC-4 items	“Strongly disagree”	“Disagree”	“Neither agree or disagree”	“Agree”	“Strongly agree”	Cronbach’s alpha if item deleted	Correlation with test score
Item 1	33%	32%	26%	8%	1%	0.78	0.86
Item 2	36%	38%	16%	8%	1%	0.76	0.89
Item 3	22%	26%	28%	22%	2%	0.84	0.77
Item 4	30%	28%	26%	14%	2%	0.83	0.79

### Descriptive statistics

Table 2 reports descriptive statistics for sociodemographic and occupational data with corresponding PSC-4 mean score and standard deviation. The average score of PSC was 8.98 ± 3.48. There was a prevalence of women (64.5%) and night shift workers (78%); most frequent occupational roles were nursing (41%) and physicians (39%). Among the participants, the majority (78%) experienced at least one episode of workplace violence (verbal and/or physical).

**TABLE 2:** Sociodemographic and occupational characteristics of the study sample.

	*N* (%)	PSC-4 mean score (sd)	*P*-value
Total sample	276	8.98 (3.48)	
**Gender**			
Female	178 (64.5%)	9.12 (3.61)	0.35
Male	98 (35.5%)	8.72 (3.24)	
**Age range**			
20–29	16 (6%)	8.62 (2.63)	0.80
30–39	95 (34.5%)	9.09 (3.05)	
40–49	81 (29%)	8.98 (3.72)	
50–59	66 (24%)	8.61 (3.77)	
>60	18 (6.5%)	10.06 (4.05)	
**Occupational role**			
Physicians	107 (39%)	8.29 (3.42)	<0.001
Nurses	113 (41%)	8.75 (3.09)	
Health assistants	30 (11%)	10.9 (4.42)	
Other health workers	26 (9%)	10.5 (2.11)	
**Shift worker**			
No	32 (12%)	9.59 (3.36)	0.44
Yes, with night shifts	216 (78%)	8.88 (3.45)	
Yes, without night shifts	28 (10%)	9.50 (3.73)	
**“The general violence prevention system of the Hospital is perceived as adequate”**			
No	222 (80.5%)	8.79 (3.51)	0.24
Yes	36 (13%)	9.53 (3.45)	
Missing	18 (6.5%)		
**Experienced episodes of workplace violence**			
None	60 (22%)	9.00 (3.44)	0.37
<3	50 (18%)	9.38 (3.20)	
3–10	87 (31%)	9.28 (3.41)	
11-20	19 (7%)	8.95 (3.61)	
>20	60 (22%)	8.20 (3.86)	

Frequencies, means, standard deviations, and *p*-value for *t*-test (binary variables) or one-way ANOVA.

Psychosocial safety climate mean scores were associated with occupational role, with physicians reporting the lowest (8.29; *p* < 0.001) PSC value compared with colleagues.

Considering gender and age, PSC was higher among women and workers aged more than 60 years although differences were not statistically significant; similarly, night shift workers reported a lower PSC value compared with daily and other shift workers. Among workers who experienced workplace violence, PSC decreased as the number of episodes of violence increased.

Table 3 shows means, standard deviations, correlations between Job demands, Job resources, health outcomes, and PSC at both level 1 (individual level) and level 2 (hospital ward level), and intra-class coefficients (ICCs).

**TABLE 3:** Internal consistencies, means, standard deviations, correlations between study variables, and intra-class coefficients (ICCs).

	Cronbach’s alpha	Mean (sd)	Range	1	2	3	4	5	6	7	ICC
**Job demands**											
1. Emotional demands	0.79	3.1 (0.7)	1–4	1	0.42	–0.15	–0.16	0.3	–0.17	–0.21	0.14
2. Effort	0.68	16.2 (3.9)	5–25	0.34***	1	–0.30	–0.54*	0.67**	–0.45	–0.32	0.11
**Job resources**											
3. Reward	0.84	40.1 (9.1)	15–55	–0.25***	–0.45***	1	0.46*	–0.52*	0.71**	0.66**	0.09
4. Physical work environment	0.76	2.4 (0.6)	1–4.2	–0.25***	–0.34***	0.32***	1	–0.64*	0.83***	0.63**	0.22
**Psychological health**											
5. Burnout	0.63	54.6 (14.9)	20.8–87.5	0.34***	0.51***	–0.37***	–0.27	1	–0.68**	–0.49*	0.07
**Occupational health**											
6. Job satisfaction	0.79	21.0 (5.9)	6–32	–0.25***	–0.41***	0.49***	0.44***	–0.49***	1	0.71***	0.21
7. Psychological safety climate	0.84	8.9 (3.5)	4–20	–0.15*	–0.30***	0.45***	0.37***	–0.27***	0.45***	1	0.16

Correlations above the diagonal refer to the group-level data (level 2: hospital ward level); correlations below the diagonal refer to the individual-level data (level 1).

*p*-values: *< 0.05, **< 0.01, ***< 0.001.

All correlations were in the expected direction; the highest correlations were between PSC and Job resources, at both individual (*r* = 0.45 for Reward and *r* = 0.37 for Physical work environment) and group levels (*r* = 0.66 for Reward and *r* = 0.63 for Physical work environment); correlations between PSC and Job demands (both Emotional demands and Effort) were statistically different from zero only at individual level.

The ICC for PSC was 0.16, meaning that approximately 16% of its total variance was explained by differences between hospital wards. These results supported the hypothesis that PSC is a group-level construct. Variables regarding Physical work environment and Job satisfaction presented the highest ICC (0.22 and 0.21, respectively). The mean of the agreement indices was 0.81, indicating very good homogeneity in the responses within work units.

### Hypothesis testing

Psychosocial safety climate was significantly associated with Job demands and Job resources; specifically, PSC was negatively related to Effort (*b* = –0.36) and Emotional demands (*b* = –0.03) and positively related with Reward (*b* = 1.16) and Physical work environment (*b* = 0.06) (Table 4). Such results supported H1.

**TABLE 4:** Multilevel random coefficient model (adjusted for age and gender) examining the relationship between PSC and Job Demands/Resources (H1).

	Job demands				Job resources			
		
	Effort		Emotional demands		Reward		Physical work environment	
				
	*B*	SE	*B*	SE	*B*	SE	*B*	SE
PSC	–0.36***	0.07	–0.04*	0.01	1.16***	0.01	0.06***	0.01

*p*-values: *< 0.05, ***< 0.001.

Table 5 shows the direct effect of PSC on Burnout (*b* = –0.28) and Job satisfaction (*b* = 0.69) supporting H2. Job resources and Job demands resulted in a significant relationship with outcomes, which persisted even when PSC was added to the model. They confirmed their role as mediators (H3), as the indirect effects were statistically different form zero and, for each mediator, the Monte Carlo confidence interval did not contain zero.

**TABLE 5:** Multilevel random coefficient models and Monte Carlo confidence intervals supporting H2 and H3.

	*B*^	SE	Indirect effect	Monte Carlo CI
**Burnout**				
Effort	0.44***	0.05	−0.16***	(–0.22, –0.09)
Emotional demands	2.01***	0.34	−0.06*	(–0.06, –0.01)
PSC	−0.28***	0.25		
**Job satisfaction**				
Reward	0.29***	0.03	0.28***	(0.15, 0.37)
Physical work environment	3.68***	0.53	0.11***	(0.07, 0.22)
PSC	0.69***	0.13		

*p*-values: *< 0.05, ***< 0.001. ^*B* values remained significantly different from zero when PSC was added to the model (Effort on Burnout: *B* = 0.40; Emotional demands on Burnout *B* = 1.83; Reward on Job satisfaction *B* = 0.21; Physical work environment on Job satisfaction *B* = 2.25).

**FIGURE 3:**
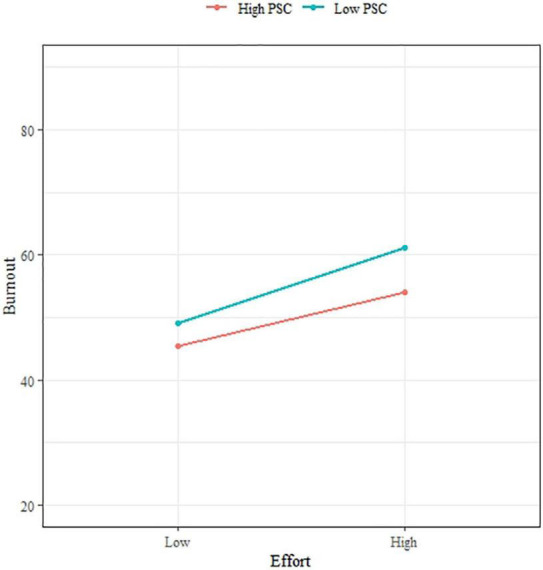
The interaction between effort and PSC on burnout.

**FIGURE 4:**
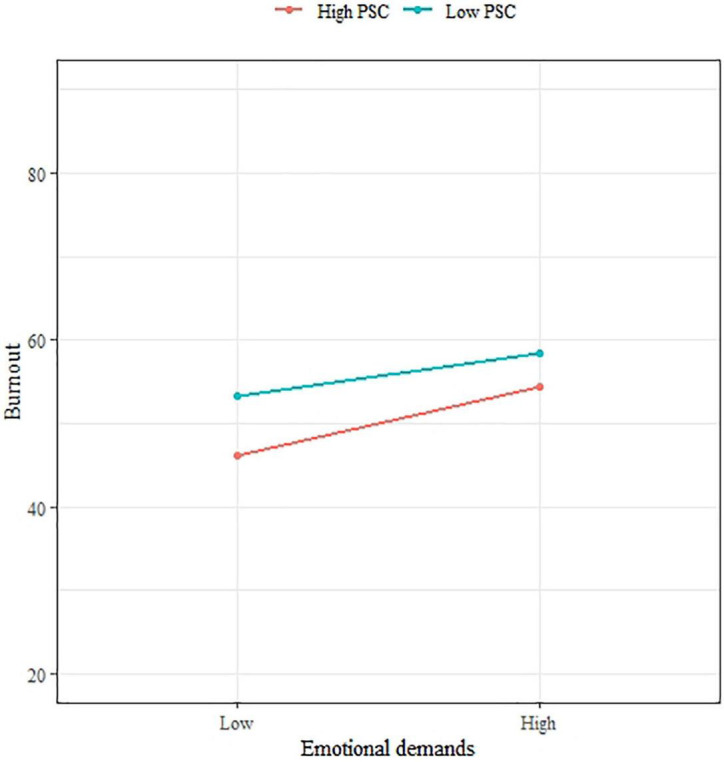
The interaction between emotional demands and PSC on burnout.

Concerning moderation, Figures 3, 4 show straight lines representing the relation between PSC and Job demands on Burnout after stratifying by different levels of PSC (high: >11, low: <12). Although the slope was slightly lower for subjects with a high level of PSC compared with those with a lower level of PSC, the interaction terms between PSC and Job demands were not significant through the multilevel analysis (Table 6). As PSC did not report as a moderator for the relation between Job demands and Burnout, H4 was not supported.

**TABLE 6:** Multilevel random coefficient model (adjusted for gender and age) to test moderation (H4).

	Burnout	
	
	*B*	SE
Effort	1.64***	0.87
PSC	–0.71	0.49
PSC × Effort	0.01	0.05
Emotional demands	1.34	0.85
PSC	–1.63	0.26
PSC × Emotional demands	–0.01	0.08

*p*-values: ***< 0.001.

## Discussion

In this study, we tested the psychometric properties of the Italian four-item version of PSC at the middle management level and examined the PSC theory in a sample of Italian healthcare workers by expanding the JD-R model. To our knowledge, this is the first study focused on the PSC framework in the Italian context.

The Italian version of PSC-4 showed good reliability, similar to the original four-item PSC (Dollard, 2019) and to the recently validated versions in Swedish (Berthelsen et al., 2020) and among United Arab Emirates (Alshamsi et al., 2022). Moreover, the ICC was 0.16, indicating that 16% of the variance in PSC could be explained by differences in the hospital wards; this result suggests that PSC is a climate construct, able to measure the perceptions shared by workers at group/unit level, thus justifying the aggregation of PSC mean values to the hospital ward level.

Coherent with the PSC theoretical framework, PSC-4 was a significant predictor of both Job demands and Job resources, supporting H1. There is evidence that PSC is a “primary cause” (or “cause of the causes”) of work stress, acting as an antecedent to work characteristics (Dollard et al., 2012a). By identifying the organizational and management practices that define job demands and resources, PSC theory supports the organizational interventions to reduce psychosocial risks by establishing organizational systems to promote healthy work conditions (Dollard and Bakker, 2010). Specifically, it suggests that management involvement is key to establish, develop, and sustain organizational policies, practices, and procedure that can protect workers’ psychological health and wellbeing, as they share responsibility for the way tasks/activities are designed (Dollard and Karasek, 2010). In our study, we tested PSC at the middle management level by asking participants to evaluate PSC in their specific hospital ward: Although we cannot exclude that workers’ opinion on the highest levels of hospital management affected their judgments on PSC, our results suggested that also middle management can play a relevant role in determining and managing job demands and resources (e.g., providing support, promoting autonomy and skill discretion, and encouraging behaviors related to psychological health). This is coherent with the literature supporting the influence of mid-leaders over the team climate as through their communication and behavior they act as role models and are able to enact organizational policies to protect employee wellbeing (Klebe et al., 2021; Loh et al., 2021).

In this respect, considering PSC as an intervention target, research has shown that middle managers can be effectively trained to build and increase PSC with a significant change in PSC levels within 4 months (Dollard and Bailey, 2021); these findings also showed that PSC interventions helped middle managers in facing major stressful event such as COVID-19 and the associated work demands.

Just as midlevel leaders are essential resources to enact PSC principles in their team, it is also fundamental to sustain their psychological health as research has strongly pointed out the association between leaders negative wellbeing (e.g., high burnout) and destructive leadership style (Kaluza et al., 2020); organizational context with low level of PSC may influence line managers burnout which in turn can reduce their managerial quality and ability to enact PSC practices in their team (Parent-Lamarche and Biron, 2022).

We found a direct effect of PSC on Burnout and Job satisfaction, supporting H2. As shown by previous studies, PSC can predict psychological and organizational outcomes and this association was shown also in longitudinal studies with cross-level effects of PSC (measured at Time 1) on emotional exhaustion and psychological distress (measured from 3 to 24 months later at Time 2) (Law et al., 2011; Dollard et al., 2012a; Idris et al., 2012; Idris and Dollard, 2014). The results also sustained the extended motivational pathway as Job resources and Job demands mediated the relationship between PSC and Job satisfaction/Burnout, respectively.

In our study, PSC did not moderate the impact of negative work conditions on psychological health (Dollard et al., 2012b; Hall et al., 2013). Contrary to our findings, research has largely shown the buffering role of PSC in moderating the impact of psychosocial risk factors on wellbeing outcomes. Our results may be explained by high levels of both Burnout and Emotional demands (75% of subjects had a mean score equal or higher to 3 in a 1–5 range) and low level of PSC in our sample. Indeed, we found a lower PSC mean value compared with previous studies conducted in different occupational settings where PSC-4 was adopted (Dollard, 2019; Berthelsen et al., 2020); higher levels of PSC were also found in other studies with PSC-12 among healthcare workers (Zadow et al., 2017; McLinton et al., 2019). According to PSC Benchmark Standards, the mean PSC value found in our study suggests a high-risk level (Dollard and Bailey, 2019). Although none of these studies concerned the Italian context, making comparison difficult, it is noteworthy that the mean PSC score in our sample was also lower than those found in previous Italian studies among workers involved in palliative cares (Fattori et al., 2022) and healthcare workers facing COVID-19 (Fattori et al., 2021).

Lower scores compared with Australian standard benchmarks were also found in China and Iran, suggesting social and political effects on PSC (Afsharian et al., 2016; Pien et al., 2019). However, further research is needed to explore the role of national culture on the perception of PSC.

This study has several limitations. Cross-sectional designs limit causal claims; the prevalence of night shift workers may make results not generalizable to the overall healthcare sector; common method bias is a possible consequence of self-reported measures with effects likely in cross-sectional designs; high levels of emotional demands and emotional exhaustion together with low level of PSC may define our sample as particular, thus limiting generalizability of findings. Additionally, the results were related to PSC perceived at the middle management level and may be limited to only public sector healthcare professionals and those working in large hospitals; although previous studies conducted in other national contexts successfully tested the PSC theoretical framework among different occupational settings (e.g., police station, school teachers, private sector organizations; Zadow et al., 2019), further studies should evaluate the transferability of our results to other Italian occupational settings.

Despite limitations, the current study supports the Italian version of PSC-4 as a valid tool to evaluate PSC and its role as leading indicator of both work conditions and health outcomes.

Given its brevity, the Italian version of PSC-4 is a parsimonious measure that meets the needs of cost and time reduction when conducting organizational surveys and interventions (mainly the cost of employees’ time to complete the survey and cost of administering surveys; Dollard, 2019). From a practical perspective, it is an accurate instrument that could be used to conduct regular evaluations of psychosocial safety climate in the workplace as well as to assess the effectiveness of interventions to change PSC by addressing all levels of prevention (job design, middle management leadership and support, organizational development, senior management values, and commitment for the long-term protection of psychological health). Previous studies in different national contexts proved it is recommended to adopt a psychosocial safety climate framework as guidance for organizational intervention and continuous monitoring and also for identifying and targeting strategic interventions for each specific sector (e.g., more interaction with senior management and increasing feelings of trust in the healthcare sector; McLinton et al., 2018).

## Data availability statement

The raw data supporting the conclusions of this article will be made available by the authors, without undue reservation.

## Ethics statement

Ethical review and approval was not required for the study on human participants in accordance with the local legislation and institutional requirements. The patients/participants provided their written informed consent to participate in this study.

## Author contributions

AF: study conception and design, data interpretation, and manuscript writing. AC: data analysis and interpretation and manuscript writing. MB: study supervision and results interpretation. LB: data acquisition and results interpretation. MD: results interpretation and manuscript critical revision. All authors read and approved the final version of the manuscript.

## Appendix 1

**Table:**

PSC-4 original version	Strongly disagree	Disagree	Neither agree or disagree	Agree	Strongly agree
Senior management shows support for stress prevention through involvement and commitment					
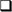
Senior management considers employee psychological health to be as important as productivity					
There is good communication here about psychological safety issues which affect me					
In my organization, the prevention of stress involves all levels of the organization					

**PSC-4 Italian version**	**Totalmente in disaccordo**	**In disaccordo**	**Né in accordo né in disaccordo**	**D’accordo**	**Totalmente d’accordo**

I miei Responsabili/Dirigenti/La mia unità operativa dimostrano/dimostra di sostenere la prevenzione dello stress con impegno ed interesse.					
La mia organizzazione/La mia unità operativa considera la salute psicologica dei lavoratori importante tanto quanto la produttività.					
Nella mia organizzazione/Nella mia unità operativa posso parlare tranquillamente delle problematiche di salute e sicurezza psicologica che mi riguardano					
Nella mia organizzazione/Nella mia unità operativa la prevenzione dello stress coinvolge tutti i livelli organizzativi.					
